# Concordance analysis of microarray studies identifies representative gene expression changes in Parkinson’s disease: a comparison of 33 human and animal studies

**DOI:** 10.1186/s12883-017-0838-x

**Published:** 2017-03-23

**Authors:** Erin Oerton, Andreas Bender

**Affiliations:** Centre for Molecular Informatics, Department of Chemistry, University of Cambridge, Cambridge, UK

**Keywords:** Microarray, Gene expression, Parkinson’s disease, Meta-analysis, Concordance

## Abstract

**Background:**

As the popularity of transcriptomic analysis has grown, the reported lack of concordance between different studies of the same condition has become a growing concern, raising questions as to the representativeness of different study types, such as non-human disease models or studies of surrogate tissues, to gene expression in the human condition.

**Methods:**

In a comparison of 33 microarray studies of Parkinson’s disease, correlation and clustering analyses were used to determine the factors influencing concordance between studies, including agreement between different tissue types, different microarray platforms, and between neurotoxic and genetic disease models and human Parkinson’s disease.

**Results:**

Concordance over all studies is low, with correlation of only 0.05 between differential gene expression signatures on average, but increases within human patients and studies of the same tissue type, rising to 0.38 for studies of human substantia nigra. Agreement of animal models, however, is dependent on model type. Studies of brain tissue from Parkinson’s disease patients (specifically the substantia nigra) form a distinct group, showing patterns of differential gene expression noticeably different from that in non-brain tissues and animal models of Parkinson’s disease; while comparison with other brain diseases (Alzheimer’s disease and brain cancer) suggests that the mixed study types display a general signal of neurodegenerative disease. A meta-analysis of these 33 microarray studies demonstrates the greater ability of studies in humans and highly-affected tissues to identify genes previously known to be associated with Parkinson’s disease.

**Conclusions:**

The observed clustering and concordance results suggest the existence of a ‘characteristic’ signal of Parkinson’s disease found in significantly affected human tissues in humans. These results help to account for the consistency (or lack thereof) so far observed in microarray studies of Parkinson’s disease, and act as a guide to the selection of transcriptomic studies most representative of the underlying gene expression changes in the human disease.

**Electronic supplementary material:**

The online version of this article (doi:10.1186/s12883-017-0838-x) contains supplementary material, which is available to authorized users.

## Background

Meta-analysis is a powerful technique for understanding gene expression in disease, increasing the power to identify true biological signal within noisy gene expression datasets. While most meta-analyses focus on the commonalities between studies, identifying the genes most relevant to the condition under study, meta-analysis approaches can also be used to shed light on inconsistencies between studies. Such analysis has led to the recognition of high levels of variation between published microarray studies of disease [1], calling into question the extent to which different tissues or model systems can represent gene expression in human patients. This is particularly noticeable in the context of microarray studies of Parkinson’s’ disease. Parkinson’s disease (PD) - a neurodegenerative disorder which causes the death of dopaminergic neurons in the substantia nigra, causing tremors and postural instability - has been well-studied at the level of gene expression, with numerous microarray studies available in public repositories. Several meta-analyses of PD gene expression in human patients have been carried out [2–4] on datasets of up to 14 unique studies; however, concordance between these studies has been reported to be low even when standardized analysis is applied [3–6]. It has been proposed that discordance could result from different progression of the disease at time of post-mortem [6] and differing amounts of neuronal loss between the substantia nigra (SN) and other regions of the brain - indeed, an analysis of 11 human PD microarray studies demonstrated increased convergence within the five studies using samples from the SN [3]. As well as differing expression patterns resulting from cytoarchitectural differences, there are patterns of tissue-specific gene expression in healthy tissue [7] such as in different regions of the brain [8, 9]. In diseased tissues, Dudley et al. [10] found that comparison across different tissues reduced the average concordance of disease gene expression from ~0.25 to ~0.10, although ‘the disease signal [remained] stronger than the tissue signal’.

Also highlighted by an early microarray study of PD [11] is the difference between animal models of PD (reviewed in Blesa et al. [12]) and the human condition, which is of much practical relevance for therapeutic research. These models were developed to mimic the clinical symptoms of Parkinson’s disease, and it is unclear to what extent the underlying patterns of gene expression will reflect those that take place in human PD. Studies comparing disease models to human patients have reported conflicting results: one study examined the consistency of gene expression between a mouse model of colorectal liver metastasis and human specimens, and found an overlap of 35% of differentially expressed genes, as opposed to 44% in normal liver tissue [13]. Another study of mouse models of inflammation found little transcriptomic agreement between human inflammatory conditions and their model counterparts [14], although a re-analysis of this data using different statistical methods questioned this conclusion [15]. As the use of transcriptomics becomes more prevalent in medicine and drug development, it is important to establish whether gene expression in a model system can be treated as a proxy for gene expression in the human condition.

Choice of microarray platform is another factor that can affect concordance between studies. Notably, although the cross-platform reproducibility of results from the same biological replicates may be high [16, 17], an early study of a mouse model of PD found very little concordance between Affymetrix and CodeLink platforms [18]. More recent studies in psoriasis [19] and in healthy tissues [7] still found detectable platform biases, indicating that this issue may not be resolved by the use of newer or more closely related microarray technologies. The effect of sample size on study concordance should also be considered: numerous simulation studies have found that larger sample sizes in microarray studies result in more stable differentially expressed gene lists [20, 21]; however, large numbers of high-quality brain tissue samples are not always easy to obtain [22, 23], and so it is advantageous to examine more directly the impact of sample size on concordance in this context.

Knowing how much concordance can be expected between studies carried out using different parameters will act as a measure of ‘representativeness’ of the recorded gene expression to true human PD, helping to establish whether animal models of disease are representative of the human condition at the transcriptomic level, and whether gene expression in more easily accessible surrogate tissues could be useful in PD research or diagnostics [24]. As the largest meta-analysis of PD to date, this study will analyse the effects of these four factors - species, tissue, platform, and sample size - to understand the reasons for the observed inconsistency between microarray studies of PD, aiming to eventually establish the relevance of these parameters to the representation of the human disease at the level of measured gene expression.

## Results

### Higher concordance of microarray studies within humans and within tissue groups

**Fig. 1:**
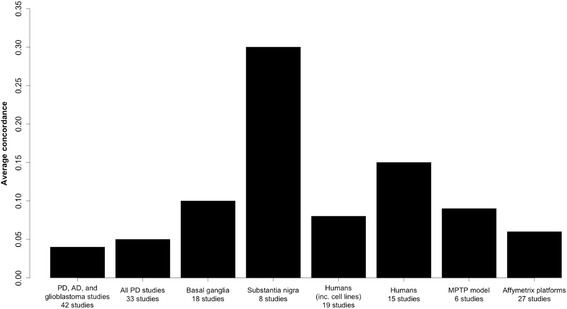
Average concordance of differential gene expression within subsets of shared factors. Average concordance over all studies is low, but increases within human patients and studies of the substantia nigra

The mean average pairwise correlation of differential gene expression signatures (i.e., the top 50 genes by absolute log fold change at a significance of *p* < 0.05, see Methods) over all 33 Parkinson’s disease studies is 0.05 (Fig. 1), indicating little overall consensus as to which genes are differentially regulated in PD. To identify how much of the observed inconsistency is due to experimental factors, concordance was examined within subgroups of studies that shared characteristics including species, tissue, or platform (Table 1, Fig. 1).

**Table 1:** Average concordance of differential gene expression signatures in microarray studies

Subset	Number of studies	Average concordance of expression signatures
PD studies plus Alzheimer’s disease and glioblastoma studies	42	0.04
All PD studies	33	0.05
Species		
Human (inc. human cell lines)	19	0.08
Human patients	15	0.15*
Mouse models	9	0.03
Rat models	4	−0.04
Disease model		
All neurotoxic models	12	0.03
MPTP	6	0.09
MPTP, mice only	5	0.10
6-OHDA	4	−0.03
Genetic models	3	0.12
Tissue		
Basal ganglia (SN (excluding isolated dopaminergic neurons) and striatum)	18	0.10*
SN: tissue	8	0.30*
SN: isolated dopaminergic neurons	4	0.03
Striatum	9	0.07
Platform		
Affymetrix	27	0.06
U133 and U133 Plus arrays (human studies only)	12	0.10

Asterisks indicate subgroups where concordance is within the top 5% of concordance values over randomly sampled subgroups of PD studies. The threshold for significance varies with the number of studies in the subset (see Methods, Additional file 12). Concordance estimates for smaller subgroups should be regarded as less reliable, as the average concordance is more sensitive to variation in individual studies

The first factor to be examined is species. The average concordance of differential gene expression signatures across experiments increases from 0.05 over all PD studies to 0.15 in human in vivo studies. In the subset of mouse studies, however, average concordance of differential gene expression decreases compared to the full dataset, at 0.03, and average concordance within the three rat studies is actually negative. This could be explained by the use of different disease models with distinct effects on gene expression: concordance within studies using neurotoxic insult to model disease is 0.09 and 0.12 for the MPTP and genetic models respectively; although there is still disagreement between studies in the 6-OHDA group (Table 1).

The next factor considered (independently of species) is the tissue type sampled. Limiting the studies under consideration to those of the basal ganglia (here including studies of the striatum and functionally also the substantia nigra), which is highly affected in PD, increases average gene-level concordance from 0.05 to 0.10, while further limiting the studies to just those of the substantia nigra yields a substantial increase to 0.30 (Fig. 1). This result is in agreement with a previous meta-analysis [3], which also reported an increase in concordance when the analysis was confined to studies of the substantia nigra. Concordance within striatal studies alone is lower than that over all tissues of the basal ganglia at 0.07; however, tissue selection is strongly associated with species, with substantia nigra studies tending to be from humans (6 of 8 studies) and striatal studies tending to be from animal models (8 of 9 studies), and so the lower concordance within the striatal group perhaps reflects the general lower concordance between animal models. To deal with issues of species dependence in tissue choice and other experimental parameters, the following analysis focuses on human studies.

### High concordance of biological pathway enrichment in human PD

**Fig. 2:**
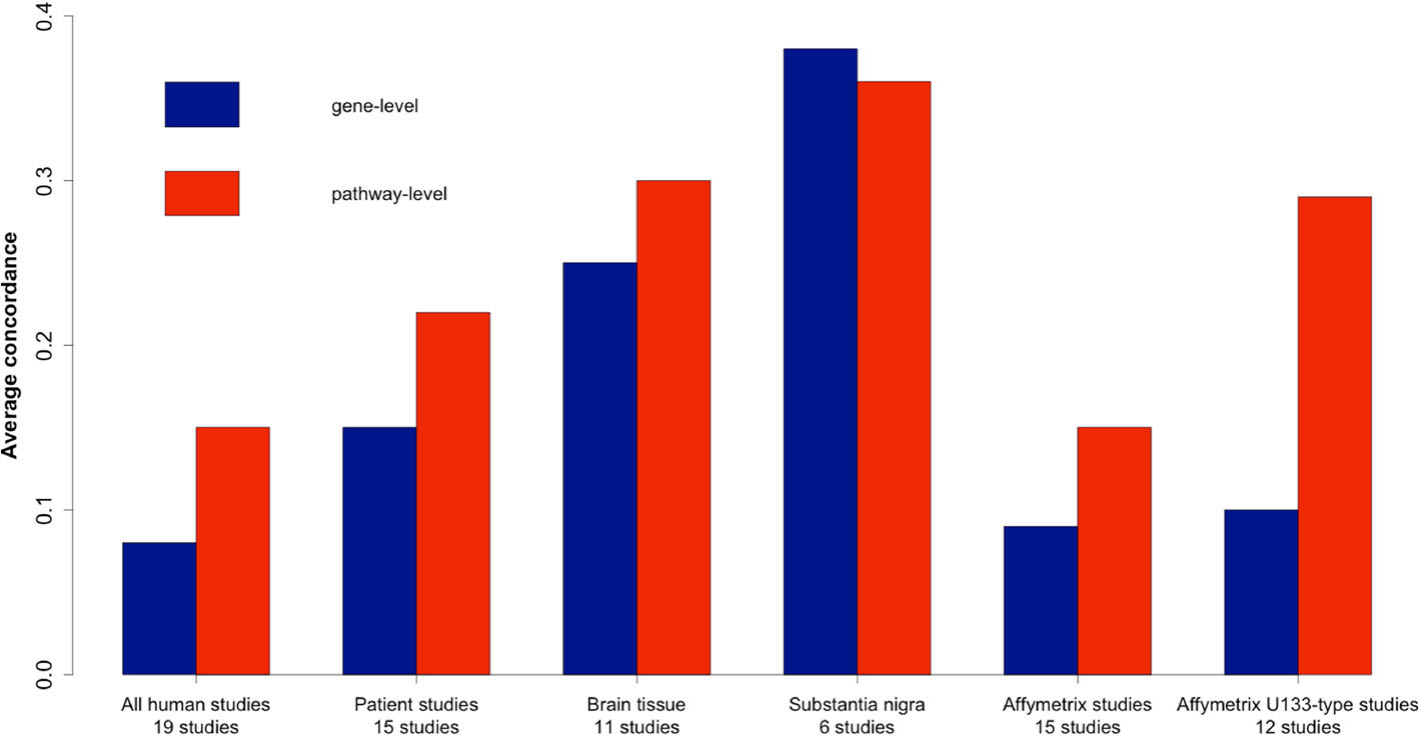
Average concordance within subgroups of human studies of PD. Concordance increases in studies of human patients (i.e., excluding human cell line studies), and within tissue subgroups. Concordance of pathways compares regulation at the level of biological processes rather than individual genes, and accordingly concordance at the pathway level is generally higher than at the level of differential gene expression

Given the low average concordance of differential gene expression, correlation was also calculated at the level of biological pathway enrichment (see Methods). As pathways are a higher-level biological concept, capturing concerted changes in the expression of several genes, we might expect to see higher concordance at this level, as demonstrated in Sutherland et al. [3] Indeed, human studies show relatively high concordance at the biological pathway level, from 0.22 over all human patient studies to 0.3 over studies of human brain tissue, indicating that measured differential expression reflects the activation of similar biological processes (Fig. 2; see Additional file 1 for a list of significant pathways). In animals, in contrast to human studies, concordance at the pathway level was in most cases actually lower than that at differential expression level (Additional file 2).

### Little effect of microarray platform on average concordance of PD studies

The next factor examined was the effect of microarray platforms, which are intended to be species-specific (one macaque study run on the U133A platform was excluded from this analysis). There is a very slight concordance increase when selecting for platform types, from 0.08 over all 19 human studies to 0.09 over all Affymetrix platforms (15 studies) and 0.10 for those studies run on the most common platform types, the Affymetrix U133A and U133 Plus series (12 studies). It should be noted that although these are different platforms, they are technically very similar, as the probe set of the U133A arrays represents a non-random subset of the U133Plus2 arrays [19], and so are considered as a single platform type for the purpose of this analysis. At the pathway level, the concordance increase within a the U133 subgroup is much larger (Fig. 2), and this may reflect the effect of a shared probeset in calculating pathway enrichment profiles, as biological pathway enrichment analysis captures concerted low-level changes in differential gene expression that are missed by the analysis of highly-regulated individual genes.

### Smaller PD studies do not show lower concordance of differential gene expression

The next factor to be examined was the study sample size. Multiple studies have found that larger sample sizes in microarray experiments allow greater confidence in calling differentially expressed genes and more robust differentially expressed gene lists [20, 21, 25], but the effect of sample size in the context of average concordance across different datasets - i.e., the likelihood of being an unrepresentative ‘outlier’ study - has not been examined directly. When the smallest 25% of human studies were excluded (excluding five studies with sample sizes of less than 10), concordance within the remaining larger studies increased slightly from 0.08 to 0.11 at the differential gene expression level and from 0.15 to 0.17 at the pathway level. Linear regression was used to test whether this implied that smaller studies were more likely to show low concordance across all (human) datasets. The association between sample size (case plus control) and average concordance of differential gene expression signatures was not significant, at a *p* value of 0.87 and an R^2^ of 0 (see Additional file 3 for plots). Similar results were obtained at the biological pathway enrichment level, (*p* = 0.93, R^2^ = 0.00, see Additional file 3 for plots).

### Visualizing the gene expression landscape of PD studies reveals a distinct subset of human studies

**Fig. 3:**
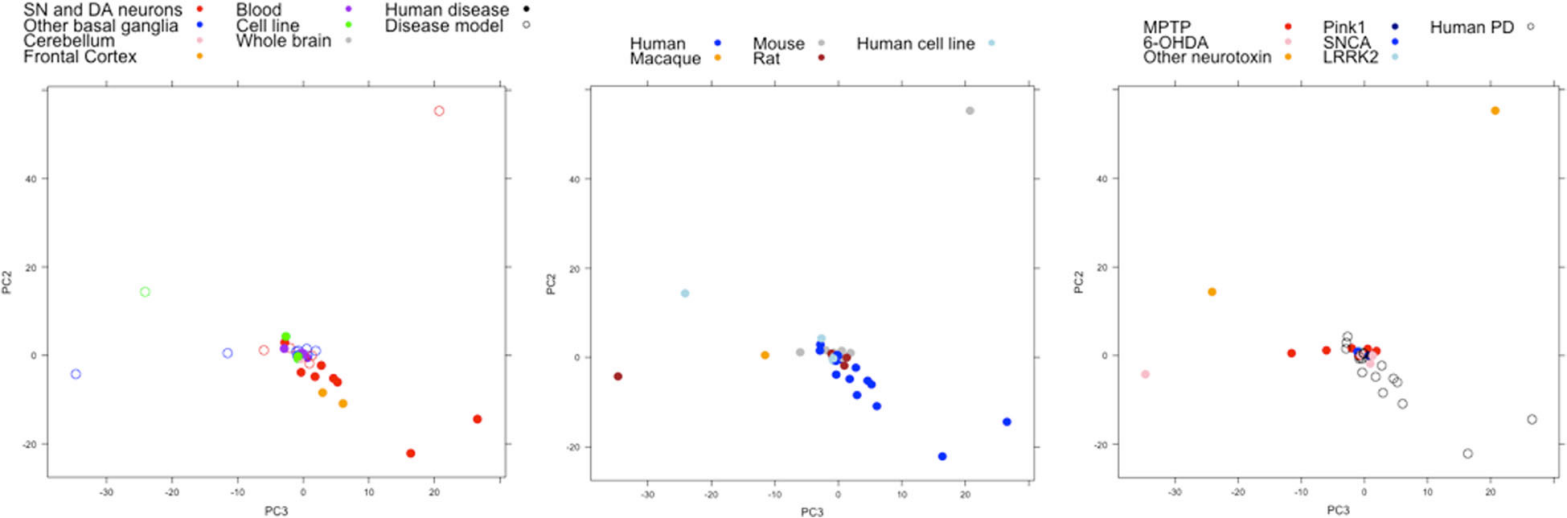
Principal component analysis of PD studies based on differential expression signatures. PCA of the 1,008 genes in the union of the top 50 genes by absolute log-fold change across all 33 studies reveals a distinct group of studies composed mainly of human studies (centre, right) of the substantia nigra and frontal cortex (left). There appears to be little separation between different disease model types (right); although the two studies using other neurotoxins (rotenone and Maneb-Paraquat) appear very distinct from the other studies. This is most clearly visualised in the second and third principal components; a similar separation is seen in the first two principal components (see Additional file 5)

The relationships between studies in differential gene expression space (here defined as the 1,008 genes in the union of expression signatures, i.e., the top 50 genes by absolute log fold change at a significance of *p* < 0.05, across all studies; see Methods; see Additional file 4 for list of 1,008 genes) were visualised using principal components analysis (PCA, Fig. 3). PCA enables representation of the 1,008-dimensional expression signature space in a lower-dimensional space which captures the greatest amount of variance amongst studies, allowing us to define a two-dimensional distance between samples which represents the correlation of their differential expression signatures [26]. The visualisation of samples in this space shows an outlying group of human studies which appear distinct from other human and animal studies (Fig. 3). This is most clearly visualised in the second and third principal components, although similar separation is seen in other components (see Additional file 5); these first three components together represent 44% of the variance.

The principal component plot is dominated by outlier studies with very large distances from the other studies in principal component space. Examination of the differential gene expression signatures of these studies (which correspond to GSE35642; GSE24233; GSE89562; GSE43490; and GSE20141; see Additional file 6) reveals that these studies show very high log fold change values in many genes, which explains their distinct position on the PCA plot. If we perform PCA on only the sign of the differential expression signatures, discarding the magnitude, the variation between studies is reduced, reducing the cluster effect but allowing clearer visualisation of the separation of studies by tissue type and species (Additional file 7).

**Fig. 4:**
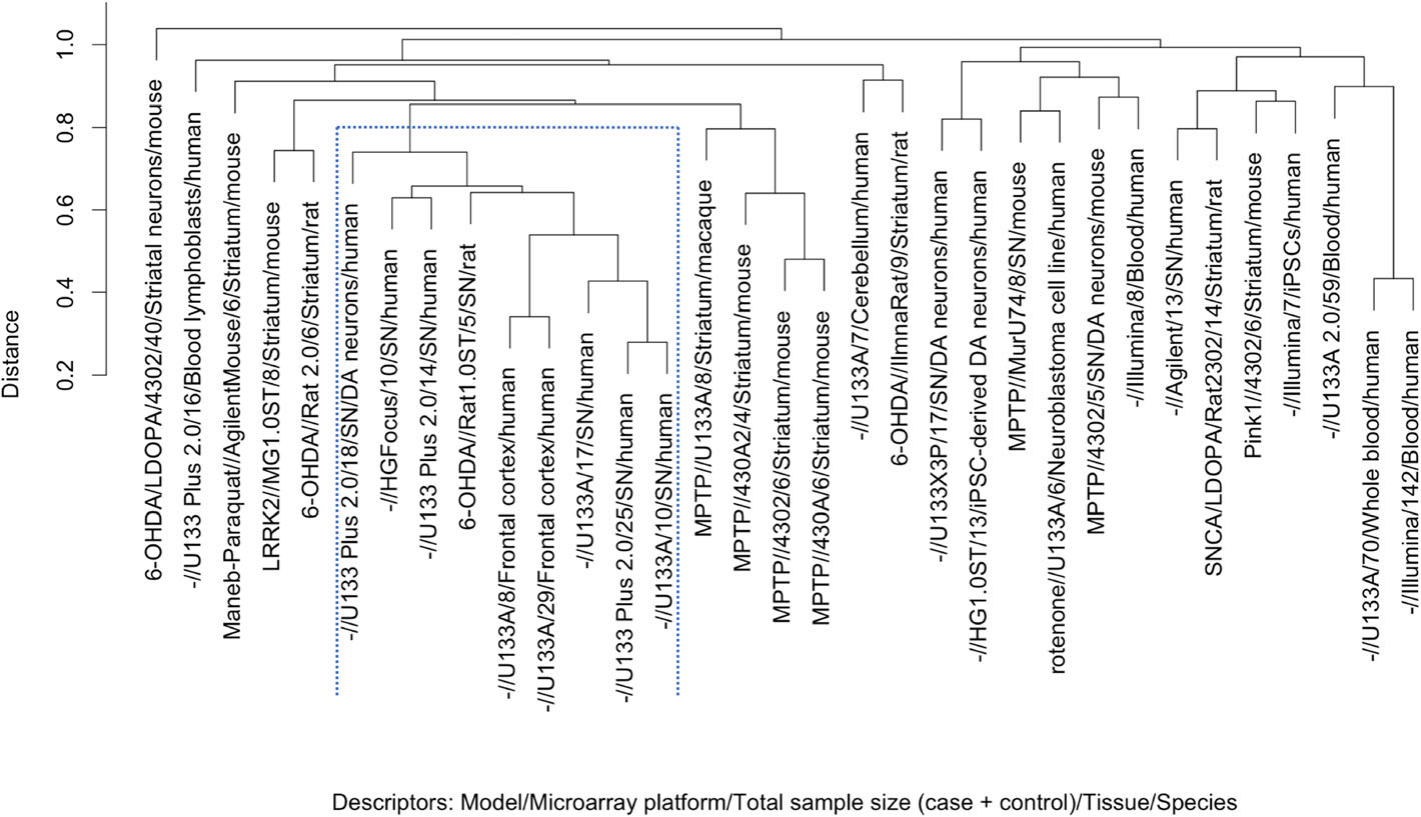
Hierarchical clustering of studies based on the most highly differentially expressed genes in each PD study. Clustering was performed based on the union of the top 10 genes by absolute log-fold change across the 33 studies. The highlighted cluster contains all but one of the human studies of the substantia nigra, as well as both human frontal cortex studies. This indicates a distinct differential gene expression pattern that is shared by these study types. This cluster also contains one rat study, however, indicating that it is possible for animal models to capture the expression patterns observed here. Aside from this outgroup, there is no apparent clustering of other factors such as platform, disease model, or treatment (e.g., with L-DOPA), reflecting the low concordance seen in these groups

In order to examine this distinct human group in more detail, hierarchical clustering was performed over the 258 genes in the union of the top 10 most differentially expressed genes all 33 studies (Fig. 4; see Additional file 4 for list of 258 genes). This shows more clearly a distinct cluster composed mainly of human studies of the substantia nigra (the most highly-affected tissue in PD) and studies of the cerebral cortex (SFG and PFC-Brodmann area 9) [27, 28], which are also affected in PD, although the cortex is affected at a later stage of disease [29]. The bootstrap *p*-value of the highlighted cluster (see Methods) is 0.99, indicating that this cluster remains highly stable under resampling of the dataset. A heatmap of the differential expression signatures (Additional file 6) reveals that studies in this cluster share downregulation in a set of genes related to protein binding and neuronal signalling (see Additional file 4 (Sheet 1) for gene names), a pattern which is not shared by animal models or other human tissues. It should be noted that a sixth study of the substantia nigra, which was run on an Agilent platform (all other studies were run on Affymetrix platforms), does not cluster, showing a distinct differential expression pattern in which the majority of genes in the expression signature are up-regulated (Additional file 6).

The clustering in Fig. 4 uses average linkage; when complete linkage is used (see Additional file 8), the SN studies form a cluster on their own, indicating that there are also expression patterns which are specific to the SN and not shared by the frontal cortex samples.

Other clusters that can be seen include 4 of the 6 MPTP models of PD, 3 of the 4 studies in blood, and clustering of iPSC studies with the appropriate tissue (dopaminergic neurons) or model (genetic animal models), although bootstrap *p*-values of these clusters are less than 95%, indicating a less stable clustering. Otherwise, there is no clear effect of any factor (such as microarray platform or treatment with L-DOPA) on study distribution within the clustering, reflecting the low concordance seen in these groups. Concordance in microarray studies of PD may therefore be partly explained by the different gene expression signals present in studies of human brains and in studies of peripheral areas or animal models.

### Differential gene expression in human tissues highly-affected in PD is distinct from other brain diseases

**Fig. 5:**
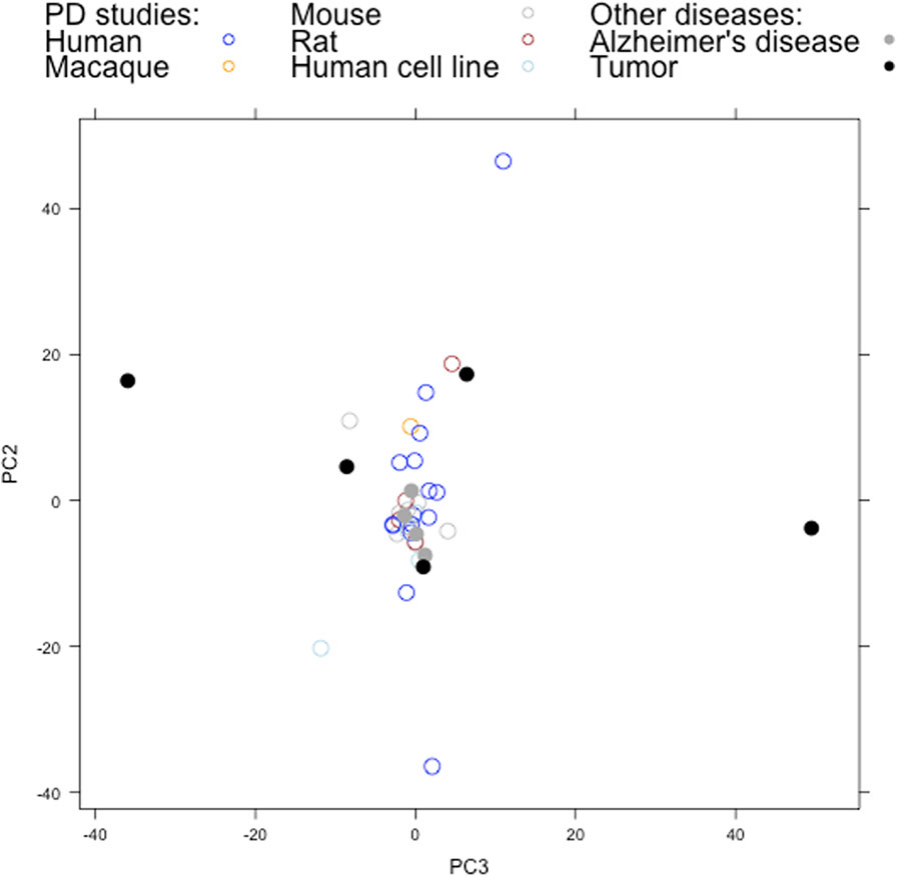
Principal component analysis of differential gene expression in Parkinson’s disease, Alzheimer’s disease and brain tumor studies. The tumor studies are mostly distant in principal component space from PD or AD studies, suggesting different patterns of gene expression in the two diseases; whilst the AD studies look very similar to those of Parkinson’s disease, suggesting that gene expression patterns in these neurodegenerative diseases could be related to some extent. This is most clearly visualised in the second and third principal components; a similar separation is seen in the first two principal components (see Additional file 13)

In order to examine the disease specificity of gene expression in PD, PD studies were clustered with studies of other diseases - namely Alzheimer’s disease (AD), a neurodegenerative disorder which can present similar pathology to PD [28], and brain tumors (glioma), which are clinically unrelated to PD. As before, PCA was used to provide a low-dimensional visualisation of the distance of samples in differential expression space; the first three principal components here represent 42% of the variance. It can be seen in Fig. 5 that while the tumor samples appear distinct in the principal component representation of gene expression space, with all but one study (a mouse tumor study using an Illumina platform) appearing separate in the PCA plot, the AD studies all cluster with PD studies, suggesting that AD gene expression studies show similar patterns of differential expression.

We can further examine these patterns using a heatmap (Additional file 9). Again, a distinctive gene expression pattern is seen for the tumor studies, while the AD studies show more similar gene expression patterns to the non-substantia nigra PD studies. This suggests that the human brain tissue group of studies shows a gene expression signal specific to PD, while the other studies may capture a more general signal of neurodegeneration.

It should also be noted that five of the six blood studies, including PD, AD, and tumor studies, cluster together on the outer edge of the heatmap, suggesting that although tumor blood gene expression pattern is still distinct from PD and AD blood gene expression, there is a signal captured in blood gene expression that is unique to these studies. Otherwise, although there are tissue differences between the tumor studies and PD studies (see Additional file 10), the distances between tumors and non-tumors are bigger than those between different tissues in PD, suggesting that the observed gene expression differences are not caused by tissue type alone.

### Inclusion of non-human and non-nigral tissue studies reduces the percentage of Parkinson’s disease-associated genes identified in a meta-analysis

A key aim of this study is to determine whether gene expression in surrogate tissue (i.e., non-brain tissue) or in animal models of disease is reflective of gene expression in the brain of a human patient. In order to establish this, a meta-analysis was carried out across different subgroups of studies, where a gene was deemed to be significant if it was included in the top 50 most highly differentially expressed genes in more than three studies (this vote-counting methodology was chosen due to the low agreement between studies; see Methods). The results of the meta-analysis were compared with a list of 694 potential PD-associated genes downloaded from the Centre for Therapeutic Target Validation [30] (see Additional file 4 for gene list). These genes were selected on the basis of previous association with PD through genetic, drug target, or text-mining association (see Methods) and represent numerous pathways including those involved in signal transduction (such as RAF/MAP kinase cascade and G alpha and AKT signalling events) and the immune system (such as interleukin-1 signalling and proteasome degradation).

The overall agreement in differentially expressed gene lists over all 33 studies was low, with no gene consistently regulated in more than 6 studies (Table 2). The most common findings include significant downregulations in genes including *ALDH1A1*, *TTR, TAC1*, and solute carrier genes *SLC18A2* and *SLC6A3*, and upregulation of the heat shock protein genes *HSPS1A* and *HSPS1B* in multiple studies. This is consistent with the findings of a previous meta-analysis [3] of human datasets, who reported concordance as low as ‘20 genes… consistently differently regulated across 6 of 13 datasets’, whilst cautioning that the downregulation seen in *DDC* and other genes could be the result of ‘a disproportionate number of SN dopaminergic neurons between cases and controls’. Other findings include downregulation of *FOS*, which is more commonly associated with overexpression following L-DOPA treatment, in two animal (non-L-DOPA treated) and one human experiments. SNCA is also downregulated in multiple human studies, which previous studies have suggested may be related to long post-mortem intervals in PD cases [31].

**Table 2:** Genes highly differentially expressed in multiple Parkinson’s disease studies. Table shows the number of times a gene is in the top 50 genes by absolute log-fold change in each study

Gene	All studies	Human studies	Studies of the SN
Up-regulated			
*HSPA1A*	4	3	3
*RELN*	4	4	3
*PTPRC*	3	2	0
*LCN2*	3	0	0
*PLIN4*	3	0	0
*MAFF*	3	2	2
*SLCO4A1*	3	3	2
*HSPA1B*	3	3	3
*IGF2BP2*	3	0	0
*CDKN1A*	3	0	0
*ENC1*	3	2	1
Down-regulated			
*EGR2*	6	0	0
*FOS*	5	2	1
*RGS4*	5	5	3
*TAC1*	5	4	3
*SLC6A3*	4	3	3
*AGTR1*	4	4	3
*FGF13*	4	3	4
*PCSK1*	4	3	2
*NPTX2*	4	1	1
*GABBR2*	4	3	2
*NR4A2*	4	3	4
*EIF1AY*	3	2	2
*SATB2*	3	0	0
*RET*	3	1	2
*SNCA*	3	3	0
*TTR*	3	0	0
*CCK*	3	0	0
*DDC*	3	3	3
*SLC18A2*	3	3	3
*ALDH1A1*	3	3	3
*KCNJ6*	3	2	2
*TMEM255A*	3	3	3
*SCG2*	3	3	3
*GPR26*	3	2	3
*DCLK1*	3	2	0
*DUSP1*	3	2	1
*HPCAL4*	3	2	1
*SYNGR3*	3	3	2
*PREPL*	3	3	0
*STMN2*	3	3	2
*VSNL1*	3	3	2
*NTS*	3	2	3

**Fig. 6:**
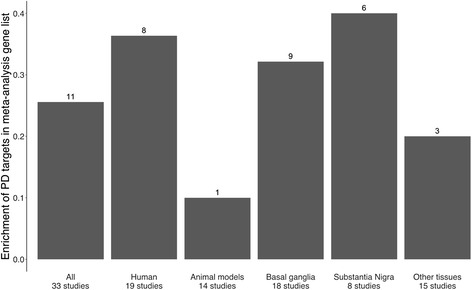
Percentage (bar) and number (number above bar) of genes previously associated with PD amongst genes identified by a meta-analysis in each grouping. Gene lists from human studies and studies using tissue from the basal ganglia (here including studies of the striatum and substantia nigra) are more enriched for genes and proteins that have been associated with PD through genetic mutations, drugs, or literature-mining than those from animal models or studies using other tissues

Over all data sets, 26% of the 43 genes called significant by our meta-analysis (Table 2) were included in the list of previously PD-associated genes. If the meta-analysis was limited to human studies, however, 36% of the 22 significant genes had previous evidence of association with PD (Fig. 6). The inclusion of non-human studies therefore reduced the enrichment of PD-associated genes in the list, i.e., the likelihood of each identified gene having a previously evidenced association with PD is lower. If the meta-analysis is limited to just animal models of PD, this was reduced to 10% of the 10 significant genes. There was a similarly noticeable difference between studies of different tissues. 32% of the 28 genes considered significant in a meta-analysis of the 18 basal ganglia studies (here including studies of the substantia nigra and striatum, excluding those which considered isolated dopaminergic neurons from the SN) had been previously associated with PD, and increasing to 40% when only substantia nigra studies were considered (Fig. 6), suggesting that gene expression in these tissue types captures changes in genes and proteins highly relevant to PD.

## Discussion

The overall concordance between microarray studies of Parkinson’s disease is low, with an average differential expression signature correlation of just 0.05, and low agreement in a meta-analysis of differentially expressed gene lists, echoing recent concerns about the reproducibility of microarray studies between different labs [3, 7, 10, 19] and between humans and animal models [11, 13–15, 32]. This study aimed to determine the major factors of study design influencing the observed lack of concordance.

The results presented here confirm that the differences between human studies and model systems, and between tissues, are larger than those caused by other experimental factors such as microarray platform or sample size (Figs. 1, 2 and 3). This analysis seems to indicate a split between human brain tissues and other study types (animal models and human studies of other tissues, including isolated dopaminergic neurons). It is possible that these human brain studies, particularly studies of the human substantia nigra, reflect a distinct ‘characteristic’ transcriptional signature specific to human PD; whereas the non-human studies and human studies of non-brain tissue reflect other, more general PD-associated molecular changes that take place in multiple tissues and systems, and are shared by other disease processes such as Alzheimer’s disease (Fig. 5). The inclusion in the ‘characteristic’ group of tissues affected later in the disease e.g., frontal cortex [29] (Fig. 4) is notable - given the progressive nature of PD, the late-affected tissues potentially display a signal of the early stages of neurodegeneration, which may be masked in the substantia nigra by the extent of cell death in this region at the time of post-mortem, as suggested by Sutherland et al. [3].

Although there are large differences between the results from animal models and human studies, it is encouraging to note that animal models (both genetic and neurotoxic) are not completely separated from human neurodegenerative disease in differential gene expression space (Fig. 3), suggesting that at least some of the underlying features of gene expression in human PD can be captured by animal models. In particular, one of the two animal models sampling tissue from the SN appears very similar to human studies in hierarchical clustering (Fig. 4), suggesting shared gene expression patterns. It is possible that these simply reflect the ‘terminal cytoarchitectural differences’ [3] related to neuronal loss in the SN; however, the observed similarity of cortical studies (neither of which show severe neuronal loss [3] compared to the SN, where next to no dopaminergic neurons remain post-mortem [28]) to studies of the substantia nigra (Figs. 3 and 4) points towards at least partly shared gene expression patterns which are reflective of other biological processes.

There is much interest in the use of non-brain tissues for gene expression studies, as these can be relatively easily obtained pre-mortem and could reflect processes associated with early-stage PD, as well as potentially offering direct patient benefit. Studies which use human cell lines, such as iPSCs derived from PD patients, do not replicate the differential expression patterns found in brain tissue but iPSC-derived dopaminergic neurons share similar expression signatures to dopaminergic neurons isolated post-mortem, while iPSCs harbouring SNCA mutations cluster with genetic animal models of PD, suggesting the ability of these study types to replicate relevant gene expression patterns in PD. Similarly, studies in blood samples cluster together, appearing distinct from gene expression in brain tissue (Fig. 4) but also appearing distinct from gene expression in blood studies of AD and brain tumors (Additional file 9), suggesting a common transcriptional pattern that could function as a PD marker. These are encouraging results for the development of these approaches for studying gene expression in PD.

## Conclusion

In practice, the concordance between microarray studies from different experimental groups will never reach 100%. Experimental factors such as array scanning and wash protocols (e.g., Ach et al. [33], van Hijum et al. [34]; reviewed in Jaksik et al. [35]) exert a significant effect on the results and reproducibility of studies; in the context of PD, there are a number of experimental factors which influence measured RNA expression in the brain including the impact of age, gender, and post-mortem interval [22, 36, 37] and other confounding factors including long-term anti-Parkinsonian drug treatment and the co-occurrence of other diseases such as Alzheimer’s disease [5]. More detailed meta-data associated with studies uploaded to public repositories would be immensely helpful in aiding meta-analysis and identification of differences between studies; both disease-specific (such as distinguishing between idiopathic and genetic PD cases, and drug-treated or drug-naïve patients) and more general (for instance, a measure of RNA integrity such as RIN [38], especially key in post-mortem studies where RNA quality is affected by the agonal state [39]).

Nevertheless, the authors believe that this study can act as a guide to the amount of agreement that can be expected between different microarray studies in the context of PD, and the general conclusions may be equally applicable in studies of other conditions. This also acts as a guide to the ‘representativeness’ of different tissues and of disease models to the human condition, which is of special significance due to the inaccessibility of PD-affected tissues in living patients, and as a guide to the use of animal models in an era of increasing use of transcriptomics and other molecular-level analyses in drug discovery and development [40]. Our identification of a specific ‘characteristic’ signal of PD in human brain tissues could explain the apparent discordance between microarray studies of PD, and is hence of more general interest for the study of PD at the transcriptomic level.

## Methods

### Obtaining Parkinson’s disease microarray studies

GEO was searched for suitable case-control studies of Parkinson’s disease using combinations of PD keywords, i.e., “Parkinson’s”/“Rotenone”/“MPTP” AND “homo sapiens”/“mammals”/“primate”, using studies submitted up to February 2017.

Inclusion/exclusion criteria were as follows:

1. Studies must be designed specifically for the investigation of PD or PD drug treatment.
2. Contrasts Parkinson’s disease (or equivalent model) versus healthy (wild-type/vehicle injected) control must be available with at least two samples for each condition.
3. Gene expression must be measured using microarray technology, as too few studies are currently available on GEO using other methods of expression profiling (such as Serial Analaysis of Gene Expression or RNA-Seq) to be able to draw any conclusions about their use in PD.
4. Human stem cell studies must be derived from PD patients and not just modelled by PD-associated mutations, in order to be comparable with human PD; equivalently, stem cells derived from PD patients compared to mutation corrected controls (such as GSE46798, GSE29773) were excluded.

This gave a total of 33 publically available studies. Four studies of Alzheimer’s disease and five studies of brain tumors (glia- and astrocyte-derived) were included as disease controls (see Additional file 10). These studies were only included in the analysis in the section ‘Differential gene expression in human tissues highly-affected in PD is distinct from other brain diseases’.

Variables recorded were the species (human, mouse, rat, or macaque); the tissue (substantia nigra, striatum, blood, frontal cortex, cell line, whole brain section, or cerebellum); the microarray platform used (Affymetrix (various types), Illumina, or Agilent); the number of cases and controls; the disease model (human PD, neurotoxic (various types), or genetic (various types)); and drug treatment status (drug treatment status was not reported for patient studies, but is known for animal models; two animal models of PD were additionally treated with L-DOPA). See Additional file 10 for details.

In order to minimise the impact of possible laboratory effects on concordance results, where multiple datasets were contributed by the same investigator and less than a year apart, only one of the two was retained (with the exception of two studies submitted as part of a meta-analysis that did not state whether the studies originated from the same experimental group, see Additional file 10). Similarly, if a single study analysed multiple tissues, only one tissue was retained for analysis. The retained study was chosen in order to provide the most balanced study design; i.e., the most even split between tissues.

### Processing of datasets

All analyses were carried out in R version 3.3.2 running under OS X 10.11.6 (El Capitan) [41]. Raw files from Affymetrix platforms were obtained from GEO and pre-processed using RMA with the Affy package, version 1.48.0 [42], or the Oligo package (REF) where necessary (for platforms Affymetrix Rat Gene 2.0 ST Array [transcript (gene) version] and Affymetrix Human Exon 1.0 ST Array [transcript (gene) version]). For experiments that used Illumina platforms, the non-normalized data was obtained from GEO, log-transformed if necessary, and quantile-normalized (for equivalence with the RMA normalization method). It is known that choice of normalization methods can affect observed correlation [43], however quantile normalization was used for this analysis as it is the standard in microarray analysis due to its use in the Affymetrix pre-processing algorithms RMA and GC-RMA [44]. For other array types (see Additional file 10), and for GSE4550 where raw data was unavailable, the submitter-supplied normalized files were used. Array quality was assessed using the ArrayQualityMetrics package [45], version 3.30.0; any samples which failed more than one of the three outlier tests (distances between arrays; boxplots; MA plots) were removed. No batch correction was used as experimental batch information is not available on GEO series records.

Log-fold change profiles were generated using limma 3.26.7 [46] as per the limma user’s guide. Probe IDs were then annotated to their associated genes using the relevant Annotation GPL file (obtained from GEO). In order to make comparisons between gene expression in different species, all non-human studies were mapped to orthologous human genes using annotationTools 1.44.0 [47]. Where multiple probes mapped to a gene, the probe with the highest *p*-value was retained. Where a probe was associated with multiple genes, the probe information was retained for both genes in order to maximise the number of genes available for comparisons between different platforms, and it should be noted that this could artificially inflate concordance between studies, especially for those using the same platform.

### Biological pathway enrichment

Biological pathway enrichment profiles were calculated from the differential gene expression profiles (generated above) against the Reactome pathway database with the GSEA function of the Bioconductor package ReactomePA 1.14.4 [48], using the default settings of 1000 permutations to calculate significance and a minimum geneset size of 10. For animal studies, the original non-ortholog genes were used to calculate enrichment profiles using mice- and rat-specific pathways provided by Reactome.

### Calculation of pairwise concordance of differential gene expression

The ‘agreement’ between two microarray studies can be measured in many different ways, including comparison of lists of genes which are differentially expressed according to some cut-off (which can be published lists, or lists created by standardized analysis of published data) [3, 5, 11], comparison of ranked gene lists [10, 49], and agreement of direction or magnitude of measured gene expression [19, 50], either over all measured genes, or over those defined as significant by some cut-off. These are reviewed in a 2009 paper by Lu et al. [51].

In this analysis, concordance between studies is defined as the Pearson correlation (as calculated by R’s cor function [52]) of their differential gene expression signatures: the 50 genes most significantly associated with the disease condition over the control condition in each study (from the set of 2,372 genes recorded by all 33 PD studies, or 2,310 over all 42 studies of brain disease, see Additional file 4 for gene lists). Log-fold change was used as the selection criteria for the signature, as it has been shown to generate gene lists of higher reproducibility compared to other ranking methods such as *P*-value ranking [25, 53, 54], so the expression signature consists of the 50 genes in each study (at a significance of *p* < 0.05) with the highest absolute log-fold change. Similar concordance results were obtained when the expression signature was defined over 20, 100, or 250 genes for each study; a value of 50 was chosen in order to capture the most relevant information while keeping the dimensionality relatively low (important in the following analyses). If correlation was calculated over the sign of the log-fold changes (i.e., considering only the direction and not the magnitude of fold changes), similar results were obtained; concordance in the SN was somewhat reduced from 0.3 to 0.22, but was still the highest-concordance tissue type, and so the measured log-fold changes were used in order to retain information.

### Calculation of pairwise concordance of biological pathway enrichment

At the biological pathway enrichment level, pairwise concordance *c*_*ij*_ between two studies was defined as the Pearson correlation of the normalized enrichment scores of pathways that are significantly up- or down-regulated (FDR <0.25, as recommended by the Broad Institute’s GSEA page [55]) in either experiment. In the case where a pathway is significant in one experiment but there is no score reported in the other, a NES of 0 was assigned for the missing pathway. If no significantly enriched pathways were reported for either experiment, the correlation was set to 0. The Pearson correlation is the most appropriate correlation measure to use given the distribution of normalized enrichment scores (a large cluster of zero-valued scores with an approximately normal distribution of the non-zero-valued scores) [56].

### Calculation of average concordances within subsets of studies

The mean of the pairwise concordances *c*_*ij*_ of a study *i* with every other study *j* in a set of studies *S* gives a measure *A*_*i*_ of how well this study agrees with other studies on average.

From the average agreement of each individual study, the average agreement *A*_*S*_ in a set can be measured (i.e., *A*_*S*_ is the average of each *A*_*i*_).

In this case, *S* is a subset of studies chosen to represent a particular factor of experimental design, such as the subset of microarray studies using human specimens, or the subset of studies run on a particular microarray platform, and the basis of this analysis is the comparison of *A*_*S*_ between these different subsets, specifically for subsets in which three or more studies shared one of the experimental factors tissue, species, platform, or sample size.

Note that in the case of differential gene expression, smaller subsets have larger numbers of shared genes, (e.g., due to sharing a platform which measures the same genes). Concordance over smaller subsets was calculated on the same expression signatures as for the set of all studies, i.e., expression signatures selected from the shared 2,372 genes, in order to ensure that *A*_*S*_ was not biased by the size of shared gene-sets in different subsets. Concordance was also calculated over the full set of genes shared by each subset, retaining a greater amount of information; results were not substantially different (see Additional file 11).

### Significance testing of subgroup concordances

Significance of average subgroup concordances was tested against the 95th percentile of the ordered distribution of average concordances over randomly sampled subgroups of the 33 PD studies (to a maximum of 100,000) of each size. An observed average correlation is significantly higher than would be expected by chance alone if it is greater than the 95th percentile value. The smaller the subgroup size, the more likely that randomly chosen subgroups show high concordance by chance alone (the distribution of observed correlations is wider), and so the confidence threshold is higher for smaller subgroups (see Additional file 12).

### Principal component analysis and hierarchical clustering

Hierarchical clustering was performed using R’s hclust function [57] using correlation distance. Correlation distance was chosen over the default Euclidean distance because it uses only the direction of gene expression changes. When Euclidean distance is used, which also uses the magnitude [58], the clustering is dominated by studies which show large log fold changes. Significance of the observed clusters was calculated using the R package pvclust [59], which uses multiscale bootstrap resampling to approximate a *p*-value for each observed cluster (*p*-values quoted are the Approximately Unbiased values). Principal component analysis was performed using R’s prcomp with centering and scaling [60]. At the differential gene expression level, the feature vector for each study was defined as its log-fold change values over the gene-set defined by the union of the 50 highest-ranking genes (the union of expression signatures; i.e., the 50 genes in each study at a significance of *p* < 0.05 with the highest absolute log-fold change) in every study in the set, in order to retain as much data as possible. For hierarchical clustering, where high dimensionality affects the stability of clusters, this was reduced to the union of the top 10 highest-ranking genes. See Additional file 4 for the lists of genes used for these analyses.

### Meta-analysis of Parkinson’s disease microarray studies

A meta-analysis over the 33 PD studies was carried out using a ‘vote-counting’ approach in which a gene was deemed to be of importance in a study if it was in the top 50 genes by absolute log-fold change, at a significance of *P* < 0.05. A gene was deemed to be significant by the meta-analysis if it was considered to be of importance by more than three studies. This threshold was chosen due to the low agreement between studies (see Results). Accordingly, only direction of association (up- or down-regulation) is reported rather than effect size. The results of the meta-analysis were compared against a list of potential PD-associated genes downloaded from the Centre for Therapeutic Target Validation [30] on 8th March 2016. This includes genes identified by genetic associations, by PD drugs, and by text-mining (see Additional file 4 for the list of genes).

The initial list downloaded from CTTV contained targets identified through reprocessing of previous RNA expression studies, which may have some overlap with those in the datasets considered here. To remove the possibility of bias resulting from potential overlap of expression studies, genes identified by RNA expression alone were excluded, leaving 694 genes from the initial list of 870. Similar results (in terms of the proportions of genes identified by each subgroup) were obtained when the meta-analysis was carried out over the top 10 or top 100 genes instead of the top 50.

## Additional files

Additional file 1: List of Reactome pathways identified as significant in human and animal studies by gene set enrichment analysis. (XLSX 12 kb)

Additional file 2: Concordance results for different subgroups using biological pathway enrichment analysis. (PDF 64 kb)

Additional file 3: Plots of average concordance against sample size for gene expression and biological pathway enrichment. (PDF 101 kb)

Additional file 4: Gene lists used for each analysis. (XLSX 154 kb)

Additional file 5: Principal component analysis of studies based on differential expression signatures, principal components 1 and 2. (PDF 110 kb)

Additional file 6: Heatmap of differential gene expression in Parkinson’s disease studies. (PDF 454 kb)

Additional file 7: Principal component analysis of studies based on sign of differential expression signatures. (PDF 190 kb)

Additional file 8: Hierarchical clustering of studies based on the most highly differentially expressed genes in each study, complete linkage. (PDF 348 kb)

Additional file 9: Heatmap of differential gene expression in Parkinson’s disease, Alzheimer’s disease, and brain tumor studies. (PDF 348 kb)

Additional file 10: GEO accession numbers of Parkinson’s disease studies included in the analysis. (PDF 121 kb)

Additional file 11: Concordance calculated over the base set of genes, consisting of the 2,372 genes recorded in all studies, vs concordance calculated over the larger sets of genes shared between subsets of studies. (PDF 72 kb)

Additional file 12: Significance thresholds of concordance for different subgroup sizes. (PDF 70 kb)

Additional file 13: Principal component analysis of differential gene expression in Parkinson’s disease, Alzheimer’s disease and brain tumors, principal components 1 and 2. (PDF 83 kb)
