# Correction to: Concordance analysis of microarray studies identifies representative gene expression changes in Parkinson’s disease: a comparison of 33 human and animal studies

**DOI:** 10.1186/s12883-019-1240-7

**Published:** 2019-01-30

**Authors:** Erin Oerton, Andreas Bender

**Affiliations:** Centre for Molecular Informatics, Department of Chemistry, University of Cambridge, Cambridge, UK

**Correction to: BMC Neurol (2017) 17:58.**

**https://doi.org/10.1186/s12883-017-0838-x**

Following publication of the original article [1], the authors reported the following errors on their article.

1) In Table 1, the ‘Average concordance of expression signatures’ of the ‘Basal ganglia’ should be 0.11^’^ instead of 0.10.

2) The rightmost bar in Fig. 2 should be 0.21 instead of 0.29. Below is the correct version of the figure.

**Fig. 2:**
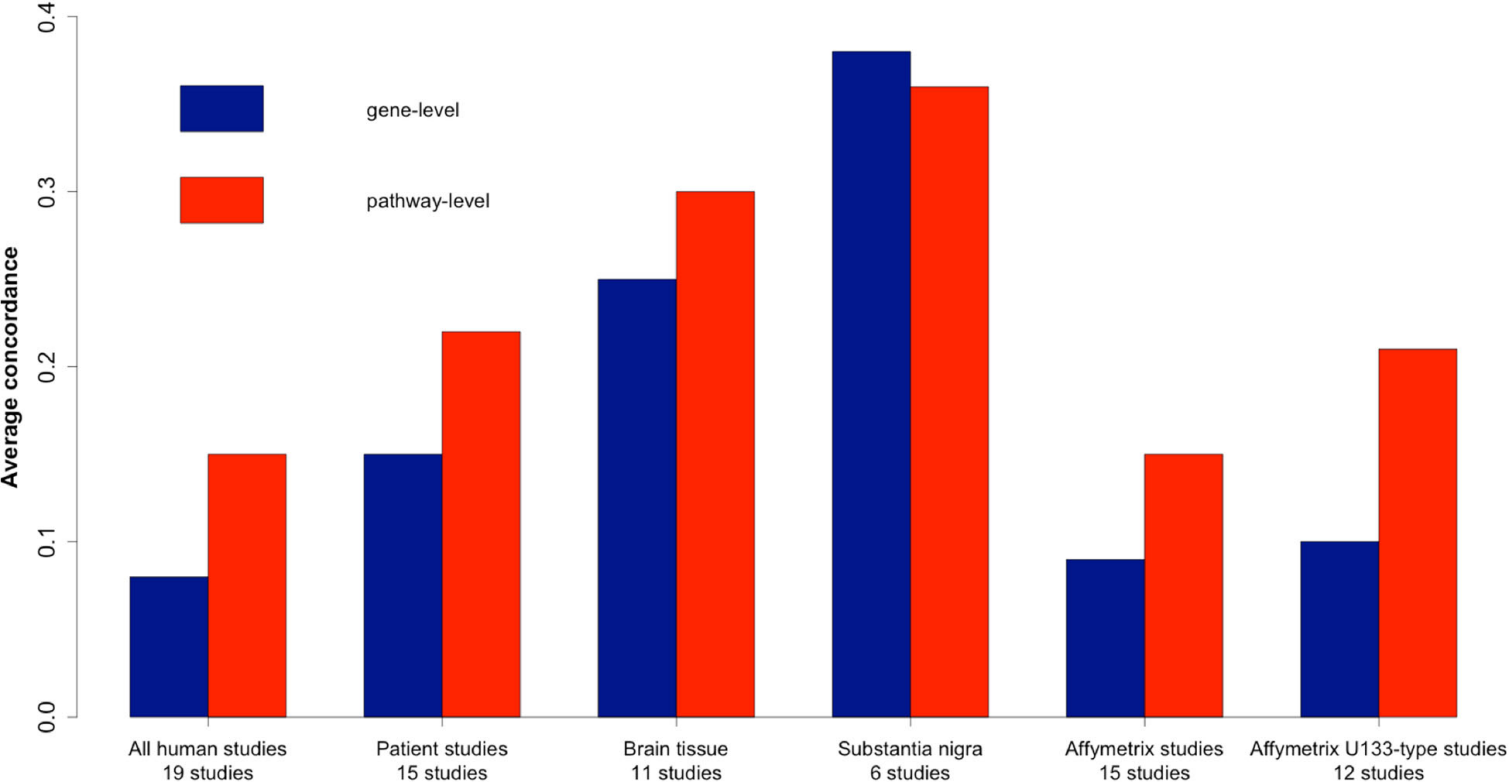
Average concordance within subgroups of human studies of PD. Concordance increases in studies of human patients (i.e., excluding human cell line studies), and within tissue subgroups. Concordance of pathways compares regulation at the level of biological processes rather than individual genes, and accordingly concordance at the pathway level is generally higher than at the level of differential gene expression
