# Supplementary material for: Concordance analysis of microarray studies identifies representative gene expression changes in Parkinson’s disease: a comparison of 33 human and animal studies
Source: BMC Neurol. 2017 Mar 23;17:58. doi: 10.1186/s12883-017-0838-x (PMC5364698; doi:10.1186/s12883-017-0838-x)
Supplement: Supplementary file 2 — Concordance results for different subgroups using biological pathway enrichment analysis. (PDF 64 kb) [file 12883_2017_838_MOESM2_ESM.pdf]

Additional file 2: Concordance results for different subgroups using biological pathway enrichment analysis.

**Subset**	**Number of studies**	**Average concordance of biological pathway signatures**
PD studies plus Alzheimer's disease and glioblastoma studies	42	0.05
All PD studies	33	0.08
Species		
Human PD	19	0.15
Human PD, *in vivo* studies only	15	0.22
Mouse models	9	0.01
Rat models	4	-0.10
Disease model		
All neurotoxic models	12	0.02
MPTP	6	0.03
MPTP, mice only	5	-0.02
6-OHDA	4	-0.01
Genetic models	3	-0.02
Tissue		
Basal ganglia (SN (excluding isolated dopaminergic neurons), striatum, globus pallidus)	18	0.10
SN: tissue	8	0.24
SN: isolated dopaminergic neurons	4	0.01
Striatum	9	0.00
Platform		
Affymetrix	27	0.09

U133 and U133 Plus arrays (human studies only)	12	0.21
