# Supplementary material for: Concordance analysis of microarray studies identifies representative gene expression changes in Parkinson’s disease: a comparison of 33 human and animal studies
Source: BMC Neurol. 2017 Mar 23;17:58. doi: 10.1186/s12883-017-0838-x (PMC5364698; doi:10.1186/s12883-017-0838-x)
Supplement: Supplementary file 3 — Plots of average concordance against sample size for gene expression and biological pathway enrichment. (PDF 101 kb) [file 12883_2017_838_MOESM3_ESM.pdf]

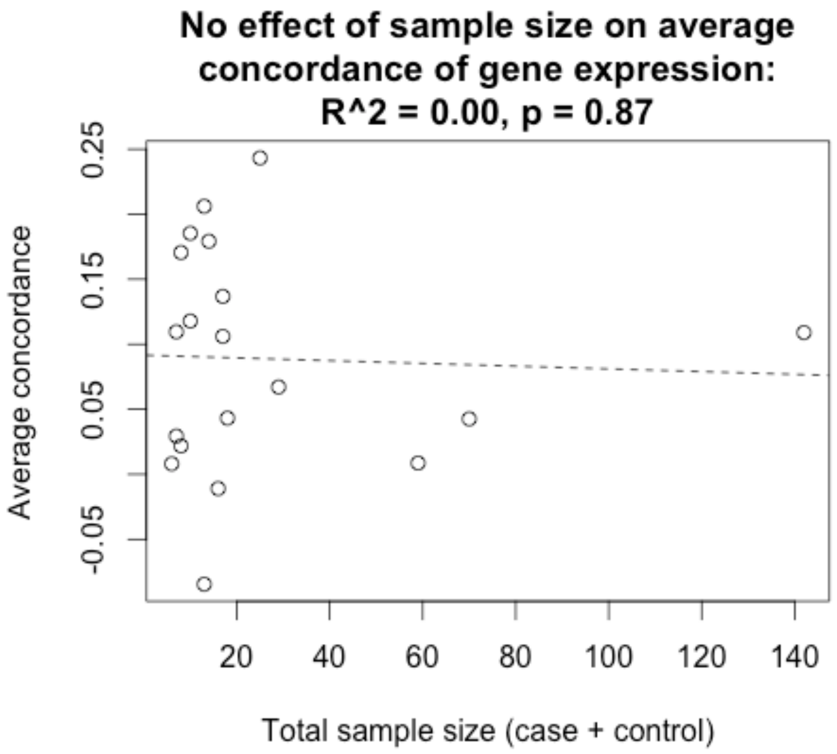

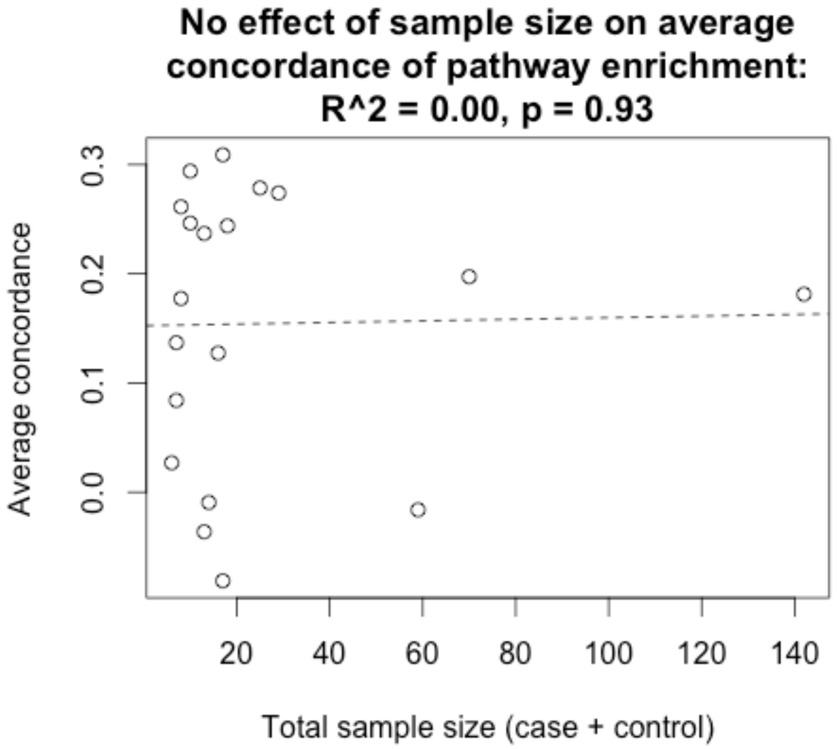

Additional file 3: **Plots of average concordance against sample size for gene expression and biological pathway enrichment.** There is no significant effect of sample size on average concordance of gene expression (top) or biological pathway enrichment (bottom).
