# Supplementary material for: Concordance analysis of microarray studies identifies representative gene expression changes in Parkinson’s disease: a comparison of 33 human and animal studies
Source: BMC Neurol. 2017 Mar 23;17:58. doi: 10.1186/s12883-017-0838-x (PMC5364698; doi:10.1186/s12883-017-0838-x)
Supplement: Supplementary file 5 — Principal component analysis of studies based on differential expression signatures, principal components 1 and 2. (PDF 110 kb) [file 12883_2017_838_MOESM5_ESM.pdf]

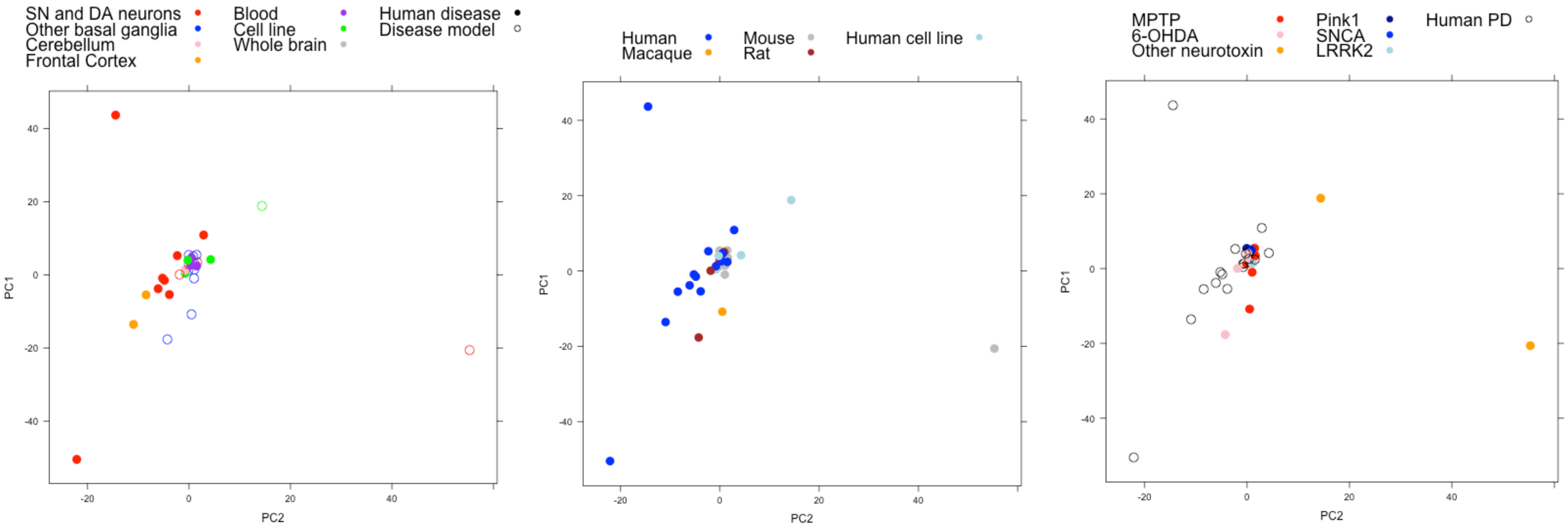

Additional file 5: Principal component analysis of studies based on differential expression signatures, first and second principal components. The distribution of studies in the first and second principal components is similar to that in the second and third principal components, showing a distinct group of human studies of the frontal cortex and substantia nigra.
