# Supplementary material for: Concordance analysis of microarray studies identifies representative gene expression changes in Parkinson’s disease: a comparison of 33 human and animal studies
Source: BMC Neurol. 2017 Mar 23;17:58. doi: 10.1186/s12883-017-0838-x (PMC5364698; doi:10.1186/s12883-017-0838-x)
Supplement: Supplementary file 6 — Heatmap of differential gene expression in Parkinson’s disease studies. (PDF 454 kb) [file 12883_2017_838_MOESM6_ESM.pdf]

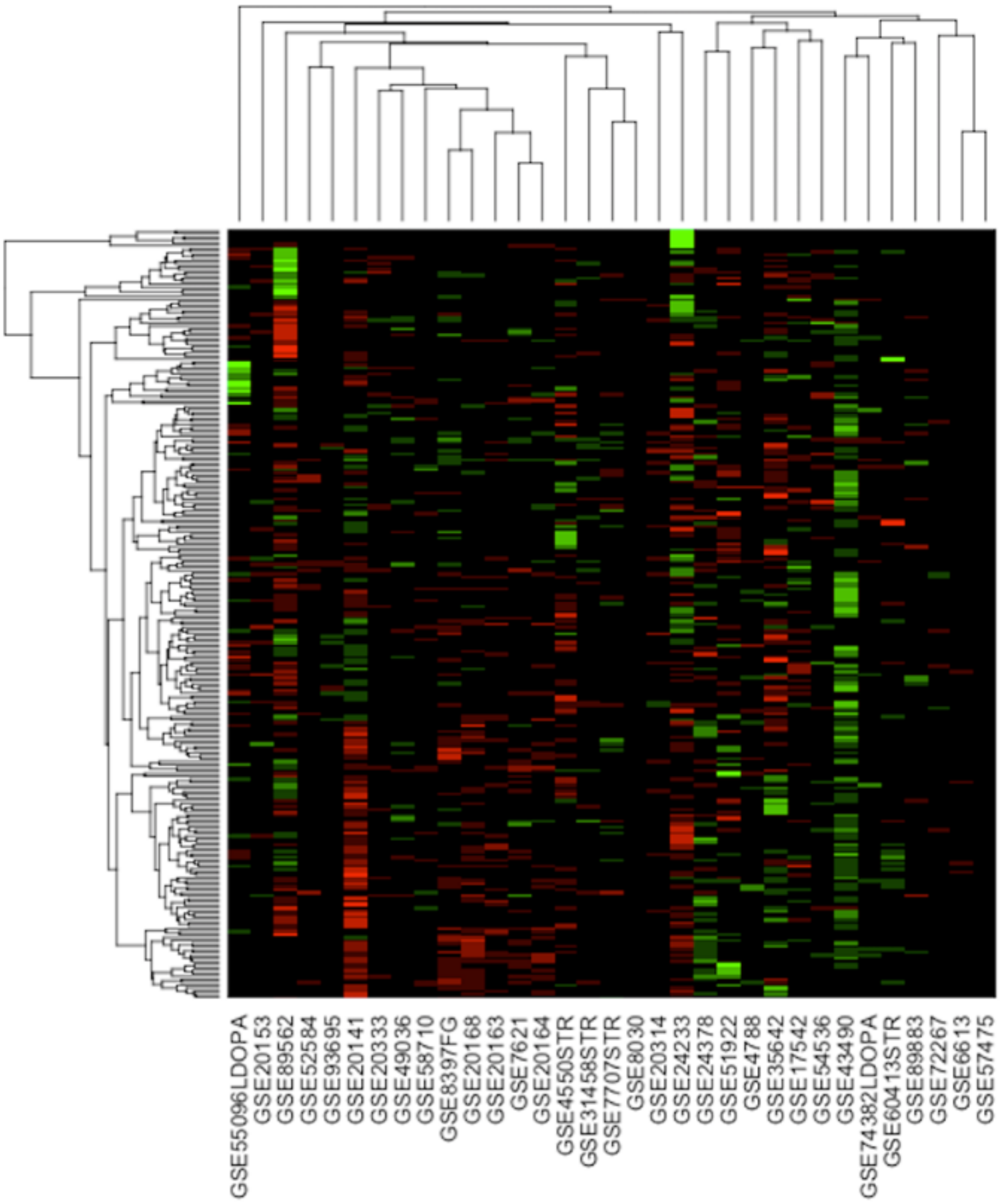

Additional file 6: Heatmap of differential gene expression in Parkinson's disease studies, showing study identifiers. Green represents upregulated genes, red represents downregulated genes. See Additional file 8 (Sheet 1) for gene names.

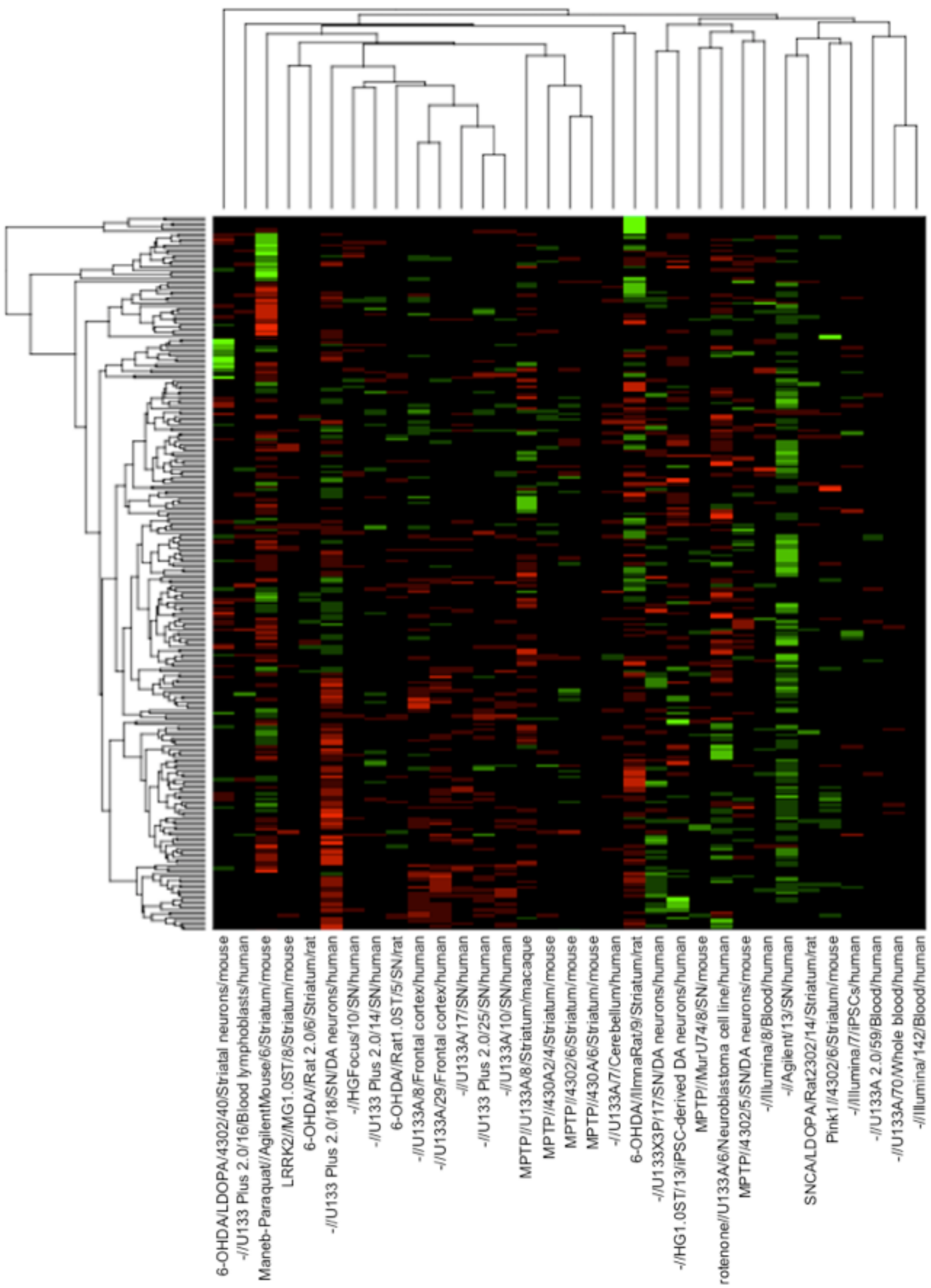

Additional file 6: Heatmap of differential expression in Parkinson's disease studies, showing study characteristics. Green represents upregulated genes, red represents downregulated genes. See Additional file 8 (Sheet 1) for gene names.
