# Supplementary material for: Concordance analysis of microarray studies identifies representative gene expression changes in Parkinson’s disease: a comparison of 33 human and animal studies
Source: BMC Neurol. 2017 Mar 23;17:58. doi: 10.1186/s12883-017-0838-x (PMC5364698; doi:10.1186/s12883-017-0838-x)
Supplement: Supplementary file 7 — Principal component analysis of studies based on sign of differential expression signatures. (PDF 190 kb) [file 12883_2017_838_MOESM7_ESM.pdf]

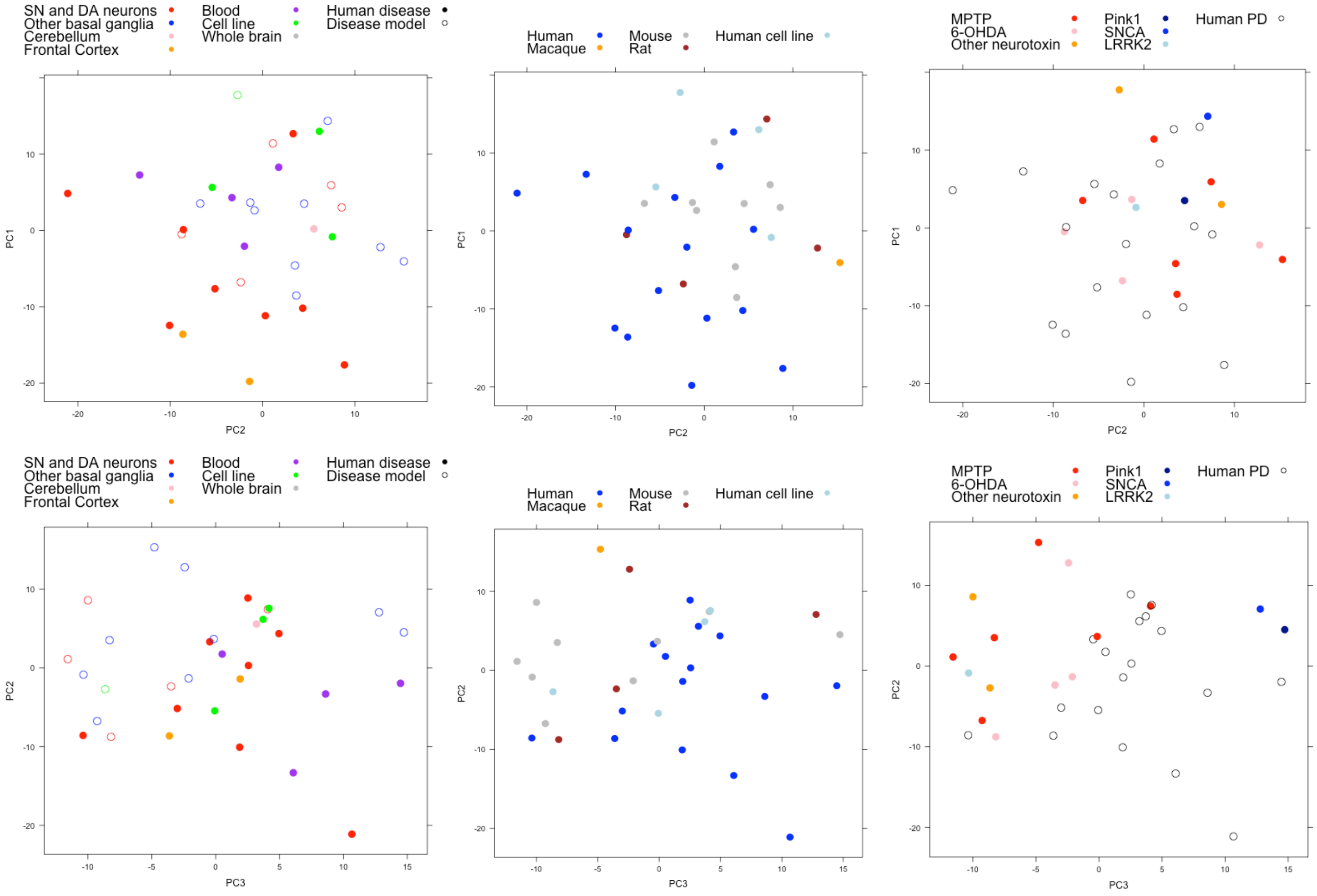

Additional file 7: Principal component analysis of studies based on sign of differential expression signatures. Separation between human and animal studies can be most clearly seen in the second and third principal components (bottom center and right). Plots of the first and second principal components have been included for comparison, and here the separation of studies seems to also reflect tissue type (top left).
