# Supplementary material for: Concordance analysis of microarray studies identifies representative gene expression changes in Parkinson’s disease: a comparison of 33 human and animal studies
Source: BMC Neurol. 2017 Mar 23;17:58. doi: 10.1186/s12883-017-0838-x (PMC5364698; doi:10.1186/s12883-017-0838-x)
Supplement: Supplementary file 8 — Hierarchical clustering of studies based on the most highly differentially expressed genes in each study, complete linkage. (PDF 348 kb) [file 12883_2017_838_MOESM8_ESM.pdf]

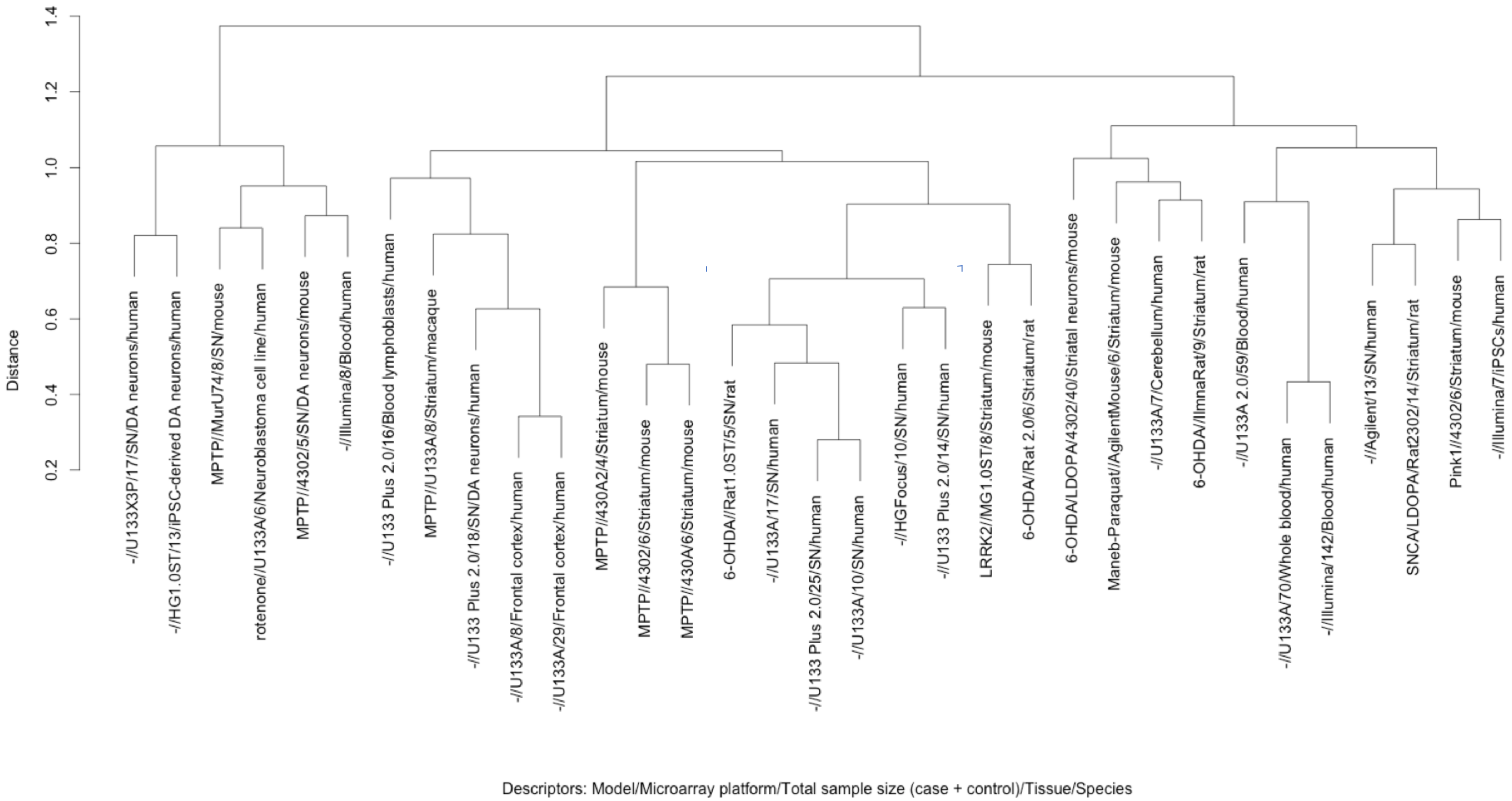

Additional file 8: Hierarchical clustering of studies based on the most highly differentially expressed genes in each study, complete linkage. Clustering was performed based on the union of the top 10 genes by absolute log-fold change across the 33 studies. When complete linkage is used, five of the six human substantia nigra studies (and one rat study) cluster separately from the other studies. The bootstrap p-value of the highlighted cluster (see Methods) is 0.96, indicating that this cluster remains highly stable under resampling of the dataset.
