# Supplementary material for: Concordance analysis of microarray studies identifies representative gene expression changes in Parkinson’s disease: a comparison of 33 human and animal studies
Source: BMC Neurol. 2017 Mar 23;17:58. doi: 10.1186/s12883-017-0838-x (PMC5364698; doi:10.1186/s12883-017-0838-x)
Supplement: Supplementary file 9 — Heatmap of differential gene expression in Parkinson’s disease, Alzheimer’s disease, and brain tumor studies. (PDF 348 kb) [file 12883_2017_838_MOESM9_ESM.pdf]

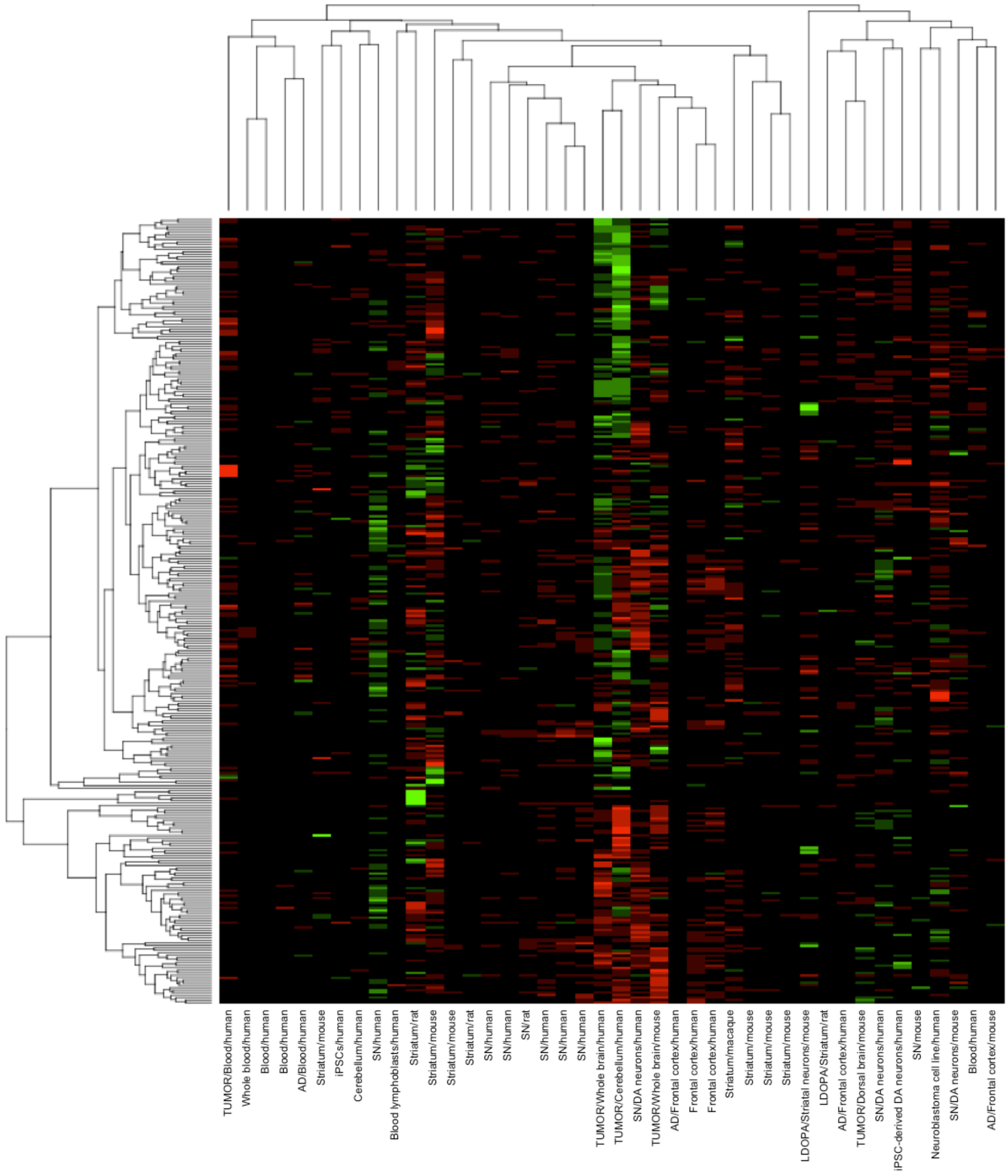

Additional file 9: Heatmap of differential gene expression in Parkinson's disease, Alzheimer's disease, and brain tumor studies. Three brain tumor studies show a distinctive pattern of upregulation of the genes at the top of the plot (see Additional file 8 for the gene names), while a fourth study of brain tumors from blood samples displays a different, but still distinct expression signature. The AD studies, by contrast, look more like the non-substantia nigra PD studies, suggesting that these studies display a common signature of neurodegeneration.
