# Supplementary material for: Concordance analysis of microarray studies identifies representative gene expression changes in Parkinson’s disease: a comparison of 33 human and animal studies
Source: BMC Neurol. 2017 Mar 23;17:58. doi: 10.1186/s12883-017-0838-x (PMC5364698; doi:10.1186/s12883-017-0838-x)
Supplement: Supplementary file 10 — GEO accession numbers of Parkinson’s disease studies included in the analysis. (PDF 121 kb) [file 12883_2017_838_MOESM10_ESM.pdf]

Additional file 10: **GEO accession numbers and study details of microarray datasets included in the analysis.**
Note: in order to minimize the impact of laboratory effects on the concordance analysis, where multiple datasets were contributed by the same investigator and less than a year apart, only one of the two was retained. However, the two studies GSE20141 and GSE20153 contributed by Middleton were first reported in the associated meta-analysis; it is not stated whether they originate from the same group so both been retained.

GEO ID	Contributor Lead author (where different) PMID (where applicable)	Submission date	Platform	Tissue	# case	# control	Species	Model	Sample selection
GSE6613	Scherzer CR 17215369	Dec 2006	U133A	Whole blood	50	22	Human	-	Parkinson's disease v healthy control; GSM153411 and GSM153454 removed as did not pass quality checks
GSE7621	Mullen JF Lesnick TG 17571925	Apr 2007	U133 Plus 2.0	Substantia nigra	16	9	Human	-	-
GSE20141	Middleton FA Zheng B 20926834	Feb 2010	U133 Plus 2.0	Laser-dissected substantia nigra pars compacta neurons	10	8	Human	-	-
GSE20153	Middleton FA Zheng B 20926834	Feb 2010	U133 Plus 2.0	B lymphocytes from peripheral blood	8	8	Human	-	-
GSE20163	Miller RM Zheng B	Feb 2010	U133A	Substantia nigra	8	9	Human	-	-

	20926834								
GSE20164	Hauser MA<br>Zheng B<br>20926834	Feb 2010	U133A	Substantia nigra	6	5	Human	-	GSM506020 removed as did not pass quality checks
GSE20168	Middleton FA<br>Zhang Y<br>15965975<br>Zheng B<br>20926834	Feb 2010; Originally published Aug 2005	U133A	Prefrontal cortex area 9 (Brodmann area 9)	14	15	Human	-	-
GSE20314	Wüllner U<br>Zheng B<br>20926834	Feb 2010	U133A	Cerebellum	4	4	Human	-	GSM509109 removed as did not pass quality checks
GSE20333	Edna G	Feb 2010	HGFocus	Substantia nigra	6	6	Human	-	GSM509556 and GSM509557 removed as did not pass quality checks
GSE24378	Cantuti-Castelvetri I<br>Zheng B<br>20926834	Sep 2010	X3P	Dopaminergic neurons isolated from substantia nigra	8	9	Human	-	-
GSE43490	Corradini BR<br>25525598	Jan 2013	AgilentPN	Substantia nigra	8	5	Human	-	SN parkinson's disease v SN control
GSE4788	Miller RM<br>15329391	May 2006	MurU74	Substantia nigra	4	4	Mouse	MPTP	MPTP MML v saline
GSE24233	Cadet JL	Sep 2010	IlluminaRat	Striatum	6	4	Rat	6-OHDA	Saline lesioned v saline control; GSM596030 removed as did not pass quality checks

GSE4550	Nahon J<br>Storvik M<br>20206263	Mar 2006	U133A	Putamen	4	4	Macaque	MPTP	Putamen MPTP day 25 v putamen saline
GSE58710	Lipton JW<br>Kanaan NM<br>25992874	Jun 2014	Rat1.0ST	Substantia nigra	3	3	Rat	6-OHDA	Wk4 6-OHDA v Wk4 vehicle; GSM1417209 removed as did not pass quality checks
GSE8030	Chin MH<br>18173235	Jun 2007	430A	Striatum	3	3	Mouse	MPTP	MPTP v control
GSE7707	Sforza DM	May 2007	4302	Striatum	3	3	Mouse	MPTP	-
GSE17542	Phani S<br>20462502	Aug 2009	4302	Dopaminergic neurons isolated from substantia nigra	3	3	Mouse	MPTP	10 day MPTP SN v control SN; GSM437382 removed as did not pass quality checks
GSE35642	Cabeza-Arvelaiz<br>22970289	Feb 2012	U133A	Neuroblastoma cell line	3	3	Human	rotenone	50nm rotenone 4 week v 0nm rotenone 4 week
GSE8397	Moran LB<br>16344956	Jul 2007	U133A	Frontal cerebral cortex - superior frontal gyrus	5	3	Human	-	-
GSE31458	Soreq L<br>22198569	Aug 2011	430A2	Striatal caudate-putamen	2	2	Mouse	MPTP	CPU MPTP FVB/N v CPU naive FVB/N<br>Note: pooled design, each 'sample' contains RNA from 3-4 of 6 mice per condition.
GSE52584	Dorval V<br>24427314	Nov 2013	MG1.0ST	Striatum	4	4	mouse	LRRK2	LRRK2 KO vs WT

GSE60413	Kurz A<br>Gispert S<br>25296918	Aug 2014	4302	Striatum	3	3	mouse	Pink1	KO 6 week v WT 6 week
GSE18309	Chen K	Sep 2009	U133 Plus 2.0	Peripheral blood mononuclear cells	3	3	human	AD	Alzheimer's disease v normal
GSE48350	Berchtold NC<br>18832152<br>(see GEO series record for other associated studies)	Jun 2013	U133 Plus 2.0	Superior frontal gyrus	21	22	human	AD	Superior frontal gyrus, age >= 70; GSM300250 removed as did not pass quality checks
GSE36980	Nakabeppu Y<br>Hokama M<br>23595620	Apr 2012	HG1.0ST	Frontal cortex	15	18	human	AD	Frontal cortex
GSE74995	Friedman B<br>Srinivasan K<br>27097852	Nov 2015	AgilentMouse v2	Whole cortex	5	5	mouse	AD	PS2APP 3 month vs WT 3 month
GSE15824	Morin PJ<br>Grzmil M<br>21406405	Apr 2009	U133 Plus 2.0	Whole brain	12	2	human	Tumor	Glioblastoma v normal brain
GSE44971	Lambert SR<br>23660940	Mar 2013	U133 Plus 2.0	Cerebellum	49	9	human	Tumor	-
GSE31095	Nilsson RJ<br>21832279	Aug 2011	AgilentFN	Blood platelets	8	12	human	Tumor	-

GSE64230	Giachino C 26669487	Dec 2014	MG1.0ST	Whole brain	4	4	mouse	Tumor	PDGF+p53-/- tumor v control
GSE57036	Sheila AL	Apr 2014	IlluminaMouse	Dorsal brain	5	5	mouse	Tumor	Tumor v dorsal control
GSE74382	Loiodice S	Oct 2015	Rat2302	Dorsal striatum	7	7	rat	SNCA	Lesion L-dopa saline vs sham saline saline
GSE55096	Heiman M 24599591	Feb 2014	4302	Striatal neurons	20	20	mouse	6-OHDA	low L-dopa 6-OHDA v saline ascorbate, Drd1a and Drd2 neurons
GSE72267	Roncaglia P Calligaris R 26510930	Aug 2015	U133A 2.0	Blood	40	19	human	-	-
GSE54536	Alieva AK 24804238	Jan 2014	Illumina HT12v4	Blood	4	4	human	-	Exclude pooled RNA
GSE93695	Chen G	Jan 2017	Rat 2.0	Striatum	3	3	rat	6-OHDA	PD v normal
GSE89562	Kumar A 27884192	Nov 2016	AgilentMouse	Striatum	3	3	mouse	Maneb-Paraquat	WT-MP v WT
GSE57475	Scherzer CR Locascio JJ 26220939	May 2014	Illumina HT12v3	Blood	93	49	human	-	-
GSE49036	Dijkstra AA 26087293	Jul 2013	U133 Plus 2.0	SN	6	8	human	-	Braak stages III and IV v control; GSM1192710_BR34_7SN removed as did not pass quality checks

GSE51922	Ezquerra M Fernandez-Santiago R 26516212	Oct 2013	HG1.0ST	iPSC-derived DA neurons	9	4	human	-	GSM1255326_SP02 removed as did not pass quality checks
GSE89883	Haenseler W	Nov 2016	Illumina HT12v4	iPSCs	4	3	human	-	Use only first clone from each patient

Abbreviations:
U133A: Affymetrix Human Genome U133A Array
U133A 2.0: Affymetrix Human Genome U133A 2.0 Array
U133 Plus 2.0: Affymetrix Human Genome U133 Plus 2.0 Array
Illumina HT12v3: Illumina HumanHT-12 V3.0 expression beadchip
Illumina HT12v4: Illumina HumanHT-12 V4.0 expression beadchip
HGFocus: Affymetrix Human HG-Focus Target Array
X3P: Affymetrix Human X3P Array
AgilentPN: Agilent Whole Human Genome Microarray 4x44K (Probe name version)
AgilentFN: Agilent Whole Human Genome Microarray 4x44K (Feature number version)
MurU74: Affymetrix Murine Genome U74A Array
IlluminaRat: Illumina ratRef-12 v1.0 expression beadchip
Rat1.0ST: Affymetrix Rat Gene 1.0 ST Array
430A: Affymetrix Mouse Expression 430A Array
4302: Affymetrix Mouse Genome 430 2.0 Array
430A2: Affymetrix Mouse Genome 430A 2.0 Array
MG1.0ST: Affymetrix Mouse Gene 1.0 ST Array
HG1.0ST: Affymetrix Human Gene 1.0 ST Array
AgilentMouse (v2): Agilent Whole Mouse Genome Microarray 4x44K (v2)
IlluminaMouse: Illumina MouseWG-6 v2.0 expression beadchip
Rat2302: Affymetrix Rat Genome 230 2.0 Array
