# Supplementary material for: Concordance analysis of microarray studies identifies representative gene expression changes in Parkinson’s disease: a comparison of 33 human and animal studies
Source: BMC Neurol. 2017 Mar 23;17:58. doi: 10.1186/s12883-017-0838-x (PMC5364698; doi:10.1186/s12883-017-0838-x)
Supplement: Supplementary file 11 — Concordance calculated over the base set of genes, consisting of the 2,372 genes recorded in all studies, vs concordance calculated over the larger sets of genes shared between subsets of studies. (PDF 72 kb) [file 12883_2017_838_MOESM11_ESM.pdf]

Additional file 11: Concordance calculated over the base set of genes, consisting of the 2,513 genes shared between all studies, vs concordance calculated over the larger sets of genes shared between subsets of studies. In most cases the result is not substantially different; concordance over the base set was reported in order not to bias the results due to changing geneset sizes.

**Subset**	**Subset size**	**Score over 2,372 genes**	**Score over shared genes**	**# shared genes**
All PD studies	33	0.05	-	2,513
SN	8	0.30	0.30	2,976
Striatum	9	0.07	0.06	6,153
Human	19	0.08	0.09	4,776
Human, in vivo	15	0.15	0.15	5,082
Mice	9	0.03	0.04	7,609
