# Supplementary material for: Concordance analysis of microarray studies identifies representative gene expression changes in Parkinson’s disease: a comparison of 33 human and animal studies
Source: BMC Neurol. 2017 Mar 23;17:58. doi: 10.1186/s12883-017-0838-x (PMC5364698; doi:10.1186/s12883-017-0838-x)
Supplement: Supplementary file 12 — Significance thresholds of concordance for different subgroup sizes. (PDF 70 kb) [file 12883_2017_838_MOESM12_ESM.pdf]

Additional file 12: Significance thresholds of concordance for different subgroup sizes.
95th percentile values of the distribution of average correlation over randomly selected subgroups of PD studies. For a correlation in any selected subgroup to be considered significant, it must be greater than or equal to the 95th percentile value for its size. As the subgroup size decreases, the spread of the distribution increases, and therefore smaller subgroups need higher average concordance to be considered as a significant deviation from the null approximation (smaller subgroups are more likely to show higher correlation through chance alone).

Subgroup size	95th percentile	Subgroup size	95th percentile
3	0.26	17	0.09
4	0.20	18	0.09
5	0.18	19	0.09
6	0.16	20	0.08
7	0.15	21	0.08
8	0.13	22	0.08
9	0.13	23	0.08
10	0.12	26	0.08
11	0.11	27	0.07
12	0.11	28	0.07
13	0.11	29	0.07
	0.10	30	0.07

14			
15	0.10	31	0.06
16	0.09	32	0.06
