# Supplementary material for: Concordance analysis of microarray studies identifies representative gene expression changes in Parkinson’s disease: a comparison of 33 human and animal studies
Source: BMC Neurol. 2017 Mar 23;17:58. doi: 10.1186/s12883-017-0838-x (PMC5364698; doi:10.1186/s12883-017-0838-x)
Supplement: Supplementary file 13 — Principal component analysis of differential gene expression in Parkinson’s disease, Alzheimer’s disease and brain tumors, principal components 1 and 2. (PDF 83 kb) [file 12883_2017_838_MOESM13_ESM.pdf]

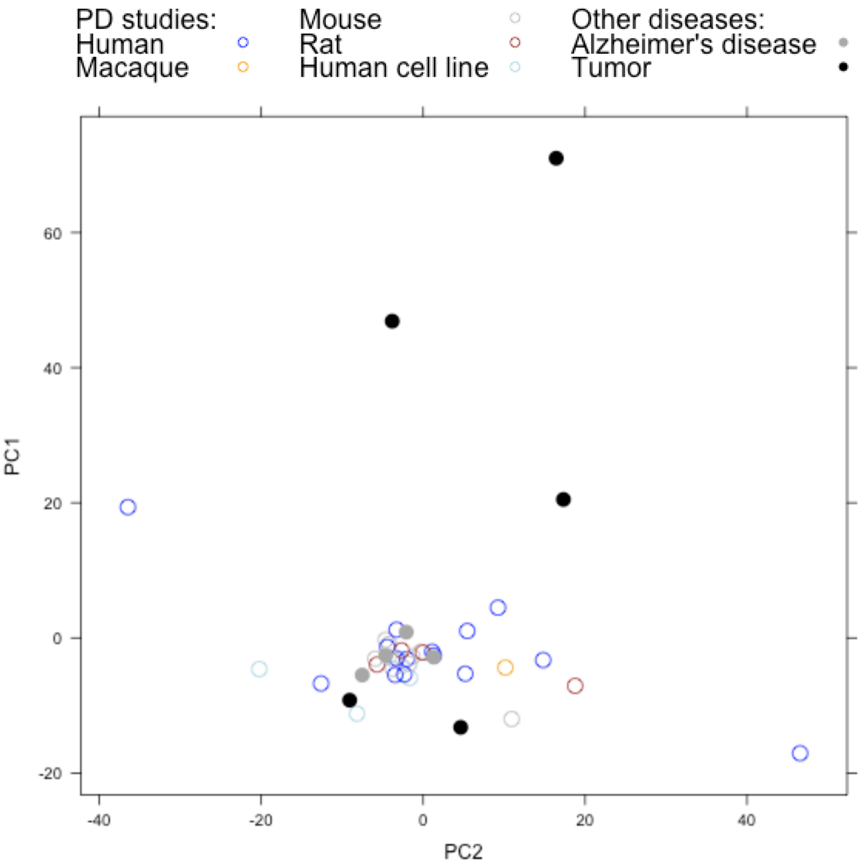

Additional file 13: Principal component analysis of differential gene expression in Parkinson's disease, Alzheimer's disease and brain tumors, principal components 1 and 2. Tumor studies are much more distant from the PD studies in principal component space than the AD studies, suggesting that neurodegenerative diseases could share some gene expression patterns.
